# Large-mode-area single-mode-output Neodymium-doped silicate glass all-solid photonic crystal fiber

**DOI:** 10.1038/srep12547

**Published:** 2015-07-24

**Authors:** Wentao Li, Danping Chen, Zhou Qinling, Lili Hu

**Affiliations:** 1 Shanghai Institute of Optics and Fine Mechanics, Chinese Academy of Science, Shanghai 201800, China; 2 University of Chinese Academy of Sciences, Beijing, China

## Abstract

We have demonstrated a 45 μm core diameter Neodymium-doped all-solid silicate glass photonic crystal fiber laser with a single mode laser output. The structure parameters and modes information of the fiber are both demonstrated by theoretical calculations using Finite Difference Time Domain (FDTD) method and experimental measurements. Maximum 0.8 W output power limited by launched pump power has been generated in 1064 nm with laser beam quality factor M^2^ 1.18.

In the high power fiber laser, the large-mode-area (LMA) single mode optical fibers have attracted much attention regarding applications^1^,^2^,^3^,^4^,^5^. In principle, LMA single-mode optical fibers can be achieved by using the conventional LMA step-index fiber. However LMA fibers require an extremely small numerical aperture to obtain single mode (SM) beam. The conventional LMA step-index fibers are difficult to fabricate with SM core diameters larger than 15 μm in a 1 μm wavelength region due to the difficulties in controlling of the refractive indices of core and cladding^6^. Since early 1996^7^, the PCFs have generated broad interest due to their unique guidance properties and applications in large mode-area structures fiber. The effective refractive index of the PCF cladding can be adjusted very precisely by controlling the air-hole diameters and the center-to-center distance between the two nearest air holes. It’s useful for designing and fabricating such fibers with extremely large core diameter values. To date, the majority of LMA-PCFs are made of silica glass with air photonic crystal cladding because of their good thermal stability and mechanical properties^8^,^9^,^10^. Soft glasses, otherwise called as multicomponent glasses, can be classified into three categories, namely, oxides, fluorides, and chalcogenides. Contrary to silica glass, soft glasses have several advantages, which are presented as follows: First, they have a wide adjustable range of refractive index, lower melting and processing temperatures (usually below 1000 °C); second, the rare earth solubility is higher than that of silica, enabling higher and more homogeneous rare-earth doping concentration^11^,^12^,^13^. Thus a shorter fiber length can be used for laser generation, which compensates the higher loss caused by the multicomponent glass compared to silica glass. Besides, compared to air hole-based PCFs, all-solid PCFs are the possibility of gluing or splicing with standard optical fibers easily. Moreover, the control of the fiber parameters during the drawing process is much simpler and the development of parameters similar to the designed ones is straightforward. Recently, there are some studies about all solid large mode area single mode phosphate glass fiber^14^,^15^, but it seems that it was difficulty to get the single mode when the fiber core diameter exceed 40 μm. As we know, it’s the first time realize a 45 μm core diameter single mode laser output in the all-solid Neodymium-doped (Nd-doped) silicate glass photonic crystal fiber.

In this paper, we designed and fabricated Nd-doped silicate glass all-solid photonic crystal with a single mode fiber laser output at the core diameter up to 45 μm.

The two kinds of un-doped silicate glass G1 and G2 of fiber cladding were bought from CDGM Glass CO., Ltd and the core Nd-doped silicate glass N0312 was made by the factory of our laboratory using melting method. Ideally, refractive index of the active core glass in a large mode area mode PCF should be equal to that of the background glass^16^. The N0312 glass was made up of SiO2, B2O3, BaO, Na2O and K2O. The proportion of the composition of BaO, Na2O and K2O were slightly adjusted to make the refractive index of N0312 equal to background glass G2 as far as possible. The rare-earth doped glass N0312 with active dopant level of 1.2 wt. %. The refractive indexes of the two kinds cladding glasses G1 and G2 were 1.50672 and 1.51230 in 1064 nm, respectively. The refractive index of the N0312 was 1.51223 in1060 nm. All refractive indices were measured using the traditional V prism method with accuracy of 5 × 10^−5^. The difference of refractive index between the background material G2 and core material is about 0.0001 at 1064nm, which was very suitable for large mode area photonic crystal fiber. The measured appropriate drawing temperatures of G1 and G2 are 810–840 °C and 820–850 °C, respectively. And the drawing temperature of N0312 was 830–860 °C. Some other physical parameters of G1, G2 and N0312 were provided in Table 1.

In order to qualify the quality of the beam emitted by an inherently slightly multimode fiber, we used the overlap factors (OF)^17^,^18^ of the guided modes in the theoretical calculation. The OF of the guided modes were computed using the following formula:
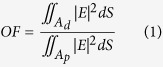


A_d_ and A_p_ represent the areas of gain region and the pump core respectively, E is the electric field distribution of the guided mode with |E|^2^ as its intensity^17^. The difference value between the overlap factor of the fundamental mode (FM) and the most confined high-order mode (HOM) was defined as ΔOF. According to previous report that a ΔOF value larger than 30% can ensure the preferential amplification and emission of the sole fundamental mode^17^. According to this theory, our fiber was theoretically investigated using Finite Difference Time Domain (FDTD) method (Mode solution V6.5 of Lumerical Corp). The diameter of the low-index G1 glass rods in the fiber cladding was d and Λ was the distance between adjacent low-index G1 glass rods. For the accuracy of the ΔOF calculation method to achieve robust single mode transmission, we calculated the OF of the FM and the first two hundred HOMs in the fiber. We got the FM and the HOM mode which has the maximum OF in the two hundred HOMs.

In Fig. 1, the two pictures were FM profile (left) and the HOM profile (right) which has the maximum OF_HOM_ in the first two hundred HOMs. Calculation results show that the fiber has single mode laser output in 1064 nm when the parameter d/Λ = 0.54, Λ = 45 μm and diameter of the fiber core was 45 μm. The OF_FM_ was 58.774% and the maximum OF_HOM_ was 27.629%, ΔOF = 31.145% > 30%, which can ensure the preferential amplification and emission of the sole fundamental mode at 1064 nm.

The fiber was fabricated using the modified stack and draw technique. First, the background material G2 glass was made into a glass tube using a mechanical processing method. According to values of theoretical calculation about d/Λ = 0.54, the inner and outer diameters of the tube are 12.42 mm and 23 mm, respectively. G1 glass rod, with a diameter of 12.42 mm, was precisely inserted into the tube to generate a composite rod. Then, the composite rod was drawn into 1 mm-diameter thin composite rods using a fiber-drawing machine. Second, the thin rods were arranged in an inner hexagonal mould to obtain a preform that is in accordance with the structure from the calculations. One rod from the core was replaced by a 1 mm Nd-doped glass rod made from N0312 silicate glass and the peripheral three rings of thin composite rods were replaced by 1 mm pure G2 glass sticks to obtain the final fiber preform. Last, the preform was drawn into the fibers. Considering the stress at the glass surface, the final cross section of the fiber is quasi circular.

Figure 2 shows the optical micrograph of the cleaved end face of a cross section of the fiber under different magnifications. The fiber cladding is composed of a periodic arrangement of low-index rods from G1 glass appear dark in a high-index background from high index G2 glass. The number of cladding rings is 5 with diameter d of the low-index rods was 24.3 μm. Corresponding to the 45 μm diameter of fiber core, the external diameter of the fiber was about 777 μm.

A 97 cm length fiber was used straightened in optical experiment to avoid the blend loss. A plane-parallel Fabry–Perot resonator was constructed to test the laser efficiency of the fiber pumped by a diode laser at a wavelength of 793 nm. The pumped laser beam from a coupled transmission fiber was collimated by an aspherical lens with a NA of 0.25 and a focus length of 11 mm. After that, the beam was focused into the fiber by another aspherical lens with a NA of 0.3 and a focus length of 6.16 mm. A dichroic mirror (Transmission 99% @793 nm, Reflectivity 99% @1064 nm) was butt-coupled to the fiber. A dichroic mirror (Reflectivity 50% @1064 nm) worked as another cavity mirror. A long wave filter with a transmission less than 0.5% at 793 nm was inserted before a spectrum meter or a power meter to make sure that only the emission light was detected. The pumping power has absorption coefficient about 10 dB/m, which measured by cut-back method. And the N0312 Nd-doped glass has 1 cm^−1^ intrinsic loss at the wavelength of 1064 nm. The fiber fluorescence spectrum was measured by a spectrum meter (Blue-Wave Miniature Spectrometers, StellarNet Inc.). The near field mode distribution across fiber facet was tested by a beam profiler (Thorlabs BP109-IR2).

Because of the single cladding structure (low coupling efficiency) and low pump power, only Maximum 0.8 W output laser in 1064 nm was achieved. The measured output laser spectrum and the output power as a function of the absorbed pumping power were shown in Fig. 3. The center wavelength of the fiber laser locates at 1064 nm and the full width at half maximum (FWHM) was 5 nm.

The near field intensity distribution of the mode displays two different profiles in the X and Y directions, corresponding to beam quality factors 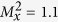 and 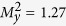 respectively. The beam quality factor M^2^ was 1.18. The Fig. 4 was the M^2^ factor measured at output power of 0.8 W.

In conclusion, we demonstrate a large mode area Nd-doped silicate glass all-solid PCF with 45 μm core diameter. The PCF was theoretical designed by FDTD method and achieved 0.8 W single mode laser output in 1064 nm with laser beam quality factor M^2^ 1.18.

## Additional Information

**How to cite this article**: Li, W. *et al*. Large-mode-area single-mode-output Neodymium-doped silicate glass all-solid photonic crystal fiber. *Sci. Rep*. **5**, 12547; doi: 10.1038/srep12547 (2015).

## Figures and Tables

**Figure 1:**
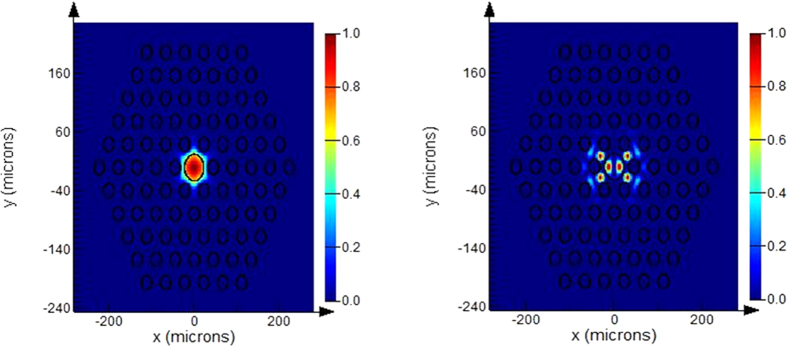
The profiles of FM (left); The profiles of HOM which has the maximum OF_HOM_ (right).

**Figure 2:**
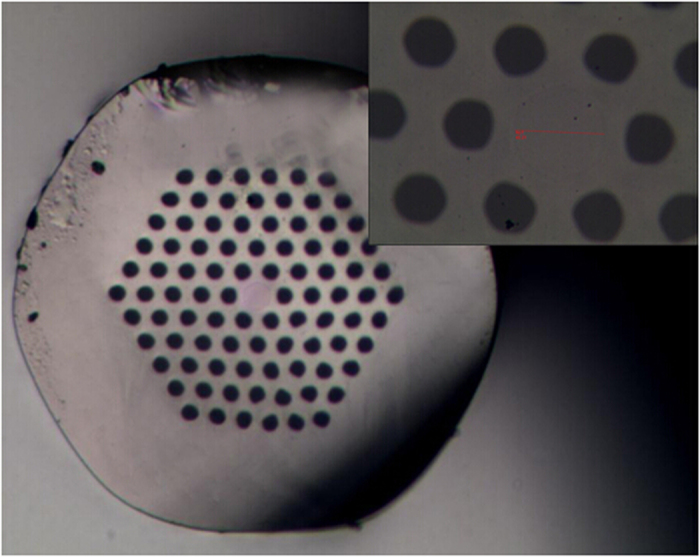
Cross section of the end face of fiber made from silicate glass under different magnification.

**Figure 3:**
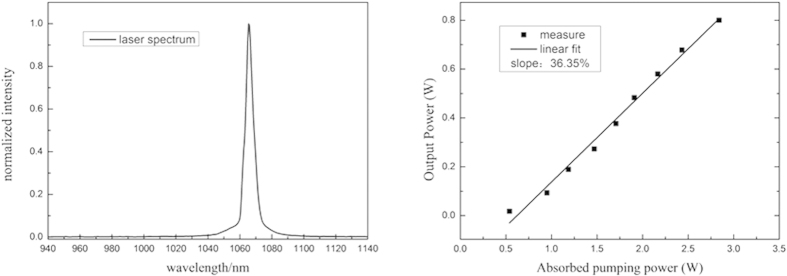
Measured spectrum of the fiber laser at an output power of 0.8 W (left); Measured output power as a function of the absorbed pumping power (right).

**Figure 4:**
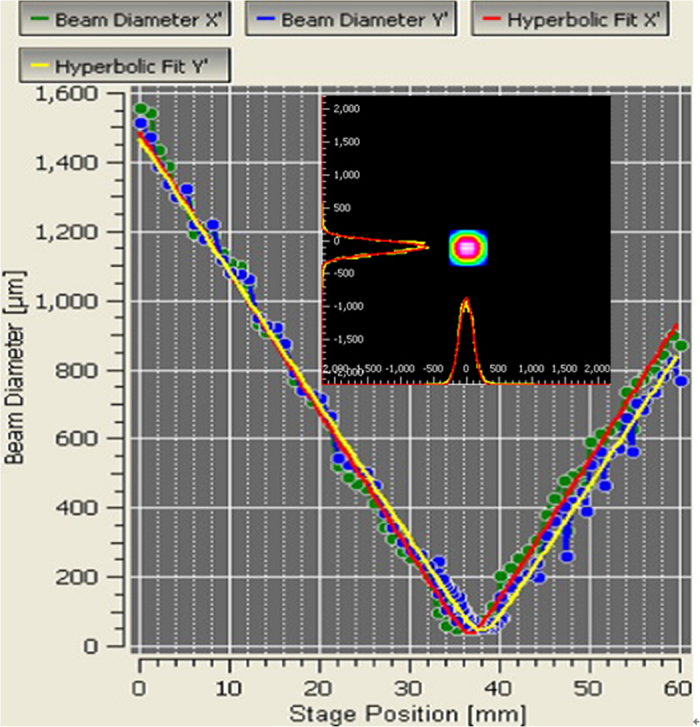
Measured M^2^ factor at an output power of 0.8 W; Inset shows mode field distribution of the fiber.

**Table 1:** 

Glass	Tg(°C)	T_10_^7.6^(°C)	α_20/120_ (10^−7^/K)	ρ(g/cm^3^)
G1	560		83	2.52
G2	564	736	82	2.52
N0312	590	660	80	2.51
Tg was the glass transformation temperature, T_10_^7.6^ was the softening point, α_20/120_ was coefficient of thermal expansion at a temperature ranges from 20 °C to 120 °C and ρ was the density of the glass at the temperature of 20 °C.				
